# Advanced Temporally‐Spatially Precise Technologies for On‐Demand Neurological Disorder Intervention

**DOI:** 10.1002/advs.202207436

**Published:** 2023-03-16

**Authors:** Xiuli Chen, Yusheng Gong, Wei Chen

**Affiliations:** ^1^ Department of Pharmacology, School of Basic Medicine Tongji Medical College Huazhong University of Science and Technology 430030 Wuhan China; ^2^ Hubei Key Laboratory of Drug Target Research and Pharmacodynamic Evaluation Huazhong University of Science and Technology 430030 Wuhan China

**Keywords:** clinical translation, neurological disorder, on‐demand, temporal‐spatial precision

## Abstract

Temporal‐spatial precision has attracted increasing attention for the clinical intervention of neurological disorders (NDs) to mitigate adverse effects of traditional treatments and achieve point‐of‐care medicine. Inspiring steps forward in this field have been witnessed in recent years, giving the credit to multi‐discipline efforts from neurobiology, bioengineering, chemical materials, artificial intelligence, and so on, exhibiting valuable clinical translation potential. In this review, the latest progress in advanced temporally‐spatially precise clinical intervention is highlighted, including localized parenchyma drug delivery, precise neuromodulation, as well as biological signal detection to trigger closed‐loop control. Their clinical potential in both central and peripheral nervous systems is illustrated meticulously related to typical diseases. The challenges relative to biosafety and scaled production as well as their future perspectives are also discussed in detail. Notably, these intelligent temporally‐spatially precision intervention systems could lead the frontier in the near future, demonstrating significant clinical value to support billions of patients plagued with NDs.

## Graphical abstract

Temporally‐spatially precise clinical intervention (TSPCI) for neurological disorders (NDs) is systematically summarized in this review. For both drug delivery and neuromodulation, the advanced strategies are illustrated to achieve temporal‐spatial precision, which can realize closed‐loop control in combination with transmissible or transmission‐free responsive systems. Typical instances as well as challenges and perspectives are provided to reveal the clinical translation values of TSPCI.

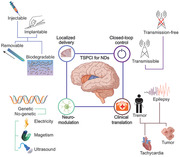

## 1 Introduction

In ingenious human nervous systems, over 14 billion neurons collaborate exquisitely and constitute intricate neural networks to perform a myriad of neurophysiological activities including sensation, motor, memory, and emotion.^[^ ^1^ ^]^ Any damage or dysfunction of neural circuits could result in undesirable neurological disorders (NDs), with typical symptoms such as paralysis, dementia, seizures, and intractable pain, which seriously compromise the life quality of suffering patients.^[^ ^2^ ^]^ The etiology and pathogenesis of NDs range from congenital genetic defects (e.g., spinal muscular atrophy^[^ ^3^ ^]^) to degenerative pathological changes (e.g., Alzheimer's Disease (AD),^[^ ^4^ ^]^ amyotrophic lateral sclerosis^[^ ^5^ ^]^). More commonly, the apoptosis and necrocytosis of fragile neurons are ascribed to various non‐neural damage factors including infection, trauma, cerebrovascular diseases (result in hypoxia of neurons), and tumors (lead to nerve invasion and compression).^[^ ^6^ ^]^ According to the Global Burden Diseases (GBD) released by Institute for Health Metrics and Evaluation, the global prevalence of NDs has been ever‐increasing over the last 30 years, reaching up to 35.7% in 2019, and directly leading to 2.2 million deaths and 65.5 million disabilities.^[^ ^7^ ^]^ Featured in high morbidity, mortality, and mutilation, NDs impose a grievous burden on healthcare systems.

Currently, drug therapy and physical neuromodulation have been recognized as mainstream strategies for the clinical intervention of NDs.^[^ ^8^ ^]^ However, their therapeutic effects are compromised to a large extent on account of the failure of temporal‐spatial precision. First, numerous NDs require long‐term or even lifelong medication with the aims of controlling unpredictable acute‐outbreak (e.g., seizure) and blocking progressive deterioration (e.g., AD), while the hindering effects of blood‐brain barrier (BBB) tremendously prevent the drugs by systematical delivery (oral intake or injection‐based administration) from approaching intracranial targets, which grievously weaken the therapeutic effects and simultaneously leave the vast majority of drugs exerting adverse effects to other parts of the body.^[^ ^9^ ^]^ Moreover, considering the genetic heterogeneity under the narrow therapeutic window, frequent therapeutic drug monitoring (TDM) is always indispensable to avoid intoxication, which is rather time and energy consuming.^[^ ^10^ ^]^ On the other side, in terms of physical neuromodulation, it could be a promising substitute or supplement for drug therapy based on the irritability of neurons and plasticity of synapses,^[^ ^11^ ^]^ and currently, transcranial magnetic stimulation (TMS) has been relatively widely accepted in clinic for the treatments of insomnia, depressive disorder, and so on, with its merits of noninvasiveness and painlessness.^[^ ^12^ ^]^ Unfortunately, the uneven skull attenuation, the mismatch between bulky machines and blurred scalp markers, as well as the traditional reliance on manual coil movement and parameter adaption, unsurprisingly impose restrictions on the accuracy of stimulation, making the exact therapeutic effects far from satisfaction.^[^ ^13^ ^]^ All these challenges prompt scientists to exploit innovative technologies with improved temporal‐spatial precision for NDs, to improve curative efficacy, reduce dosage and off‐target side effects, ensure security and convenience, and alleviate the burden of medical workers.

Notably, over the long‐term biological evolution in response to the capricious exogenous environment and dynamic physiological state of the body, the delicate nervous systems have developed inspiration‐rich strategies to satisfy temporal‐spatial precision. For instance, the electrical signals conduct fleetly through nerve fibers coated with insulated myelin to avert mutual interference,^[^ ^14^ ^]^ and the neurotransmitters are released at quantized doses with minimal diffusion between synaptic cleft to exert localized function.^[^ ^15^ ^]^ Moreover, there are a variety of receptors and effectors spreading throughout the body to gather information and executive commands respectively, cooperating closely with neural centers to form effective closed‐loop control systems with minimal time delay.^[^ ^16^ ^]^ Given the high complexity of the human neural systems, the intervention strategies for NDs must be extremely precise. Importantly, from the view of bionics, it is highly advisable to exploit neoteric intervention strategies which are qualified for playing pinpoint curative effects at lesions with negligible impact on surrounding healthy tissue. Furthermore, if combined with sensitive detection modules as well as real‐time control systems, it is feasible to trigger timely on‐demand intervention before the aggravation of illness without artificial interposition. Actualizing these assumptions is pressingly demanding, but fortunately, conquering the critical NDs and satisfying precision medicine have been set as significant goals of neuroscience. Importantly, with the mutual efforts from neurobiology, bioengineering, chemical materials, artificial intelligence (AI), inspiring step forwards in this field have been witnessed in recent years, exhibiting great clinical transformation potential.

**Figure 1:**
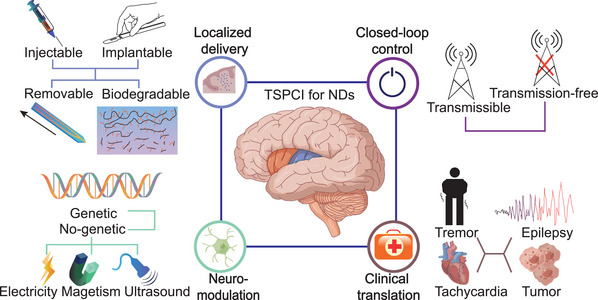
Temporally‐spatially precise clinical intervention (TSPCI) for NDs. Both drug delivery and physical neuromodulation are illustrated with an emphasis on temporal‐spatial precision. The closed‐loop control could be achieved by either transmissible or transmission‐free signal detection. The clinical translation potential is discussed with tremor, epilepsy, tachycardia, and intracranial tumor as a typical instance.

In this review, we highlight the latest progress of temporally‐spatially precise clinical intervention (TSPCI), which exerts function with localized restriction and minimal time delay, and exhibits high clinical transformation potential for NDs (**Figure** **1**). To illustrate the detailed strategies for achieving TPSCI, localized parenchyma drug delivery systems which directly target at lesions without reliance on blood or cerebrospinal fluid circulations, and precise physical neuromodulation routes which satisfy personalized desiderata with minimum disturbance to irrelevant neurons, are presented in essence. On this basis, disease‐specific biological signal detection is expounded with emphasis on promising trigger factors to realize closed‐loop control of TSPCI. Moreover, their clinic transformation potential is validated within concrete circumstances by adopting typical NDs (intracranial tumors, focal epilepsy, essential tremor, ventricular tachycardia). In addition, pervasive challenges relative to biocompatibility and scaled production as well as respective enlightening resolutions are discussed in detail to point out the future direction in this field. Arguably, these intelligent TSPCI systems could display significant clinical value in the near future to support billions of patients plagued with NDs.

## 2 Advanced Strategies for TSPCI of NDs

In recent years, multidisciplinary efforts have been made to satisfy the urgent requirements for TSPCI of NDs. In this part, localized parenchyma drug delivery and precise physical neuromodulation are systematically illustrated as the foundation for precise intervention of NDs. Subsequently, the detection of biological signals is presented in detail to trigger closed‐loop control of TSPCI.

### 2.1 Localized Parenchyma Drug Delivery

To overcome the hindering effects of BBB, plentiful researches focus on the chemical modification and parameter optimization of nanoparticles, which have been updated in the latest reviews.^[^ ^17^ ^]^ However, it should be noted that lesions of most NDs are limited within minimal parenchyma.^[^ ^18^ ^]^ Recently, the developments of chemical and engineering‐based devices are carrying through the state‐of‐art administration routines for NDs, allowing for directly targeting at parenchyma to produce high drug concentration at lesions with negligible off‐target side effects. Therefore, we herein focus on the advanced localized parenchyma drug delivery systems free from blood or cerebrospinal fluid circulations, to highlight the cutting‐edge strategies for temporally‐spatially precise drug delivery. According to whether these drug delivery systems could be absorbed in situ, they are divided into two big categories: biodegradable systems, non‐biodegradable (removable) systems.

#### 2.1.1 Biodegradable Systems

**Figure 2:**
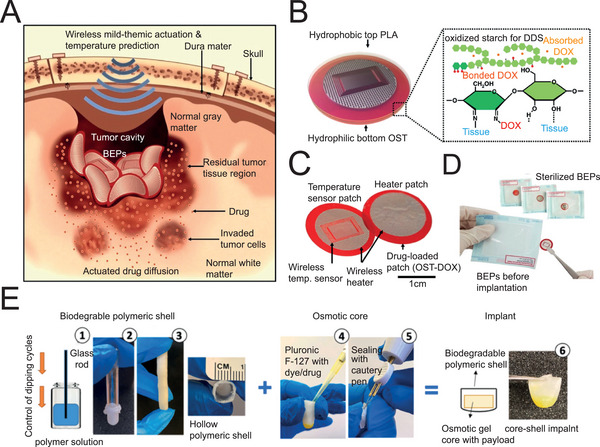
Biodegradable systems introduced by surgical implantation for localized parenchyma drug delivery. A) Schematic illustration of bioresorbable electronic patches (BEPs) which respond to the mild thermal produced by wireless RF. B) The construction of the BEP, with PLA as hydrophilic bottom, oxidized starch (OST) as hydrophobic top, and doxorubicin (DOX) as chemotherapeutic. C) Image of the BEP, which includes a bioresorbable wireless heater and a temperature sensor on an OST patch containing DOX. D) Image of the sterilized BEPs. A‐D) Reproduced under the terms of the Creative Commons CC‐BY license.^[^ ^23^ ^]^ Copyright 2019, The Authors. Published by Springer Nature. E) The procedures for fabricating the core‐shell capsules. Reproduced with permission.^[^ ^27^ ^]^ Copyright 2021, Elsevier.

Among various localized drug delivery systems, devices constituted by biodegradable polymers are the precursors targeting at parenchyma. In 1986, Gliadel, a biodegradable round wafer loaded with carmustine, was first approved by Food and Drug Administration (FDA) as the adjuvant for glioblastoma therapy via intracranial implantation.^[^ ^19^ ^]^ The spatially‐focused release of chemotherapeutics at surgical sites kills the residual tumor cell, efficaciously obviating the post‐operation metastasis or recurrence, consequently prolonging the overall survival of patients.^[^ ^20^ ^]^ Nevertheless, this controlled‐release mechanism simply depends on the natural degradation of polymers, resulting in unstable pharmacokinetics.^[^ ^21^ ^]^ An enlivening solution is fabricating core–shell nanostructure to encapsulate the active pharmaceutical ingredient (API), followed by integration into the round‐shaped wafer. The nanoscale shell functions as a rate controller to consummate long‐term and stable drug release (over 30 days^[^ ^22^ ^]^). Further improvements focus on the release process activation via transcranial physical stimulation to satisfy discontinuously on‐demand drug administration. For instance, the group of Kim synthesized a mild‐thermic responsive wafer which underwent depolymerization reaction in response to the mild temperature increment activated by a radio frequency (RF) magnetic field (**Figure** **2**A–D).^[^ ^23^ ^]^ The mild heat energy would not damage the normal white matter but accelerate the diffusion of antineoplastic drugs into deeper tissues.

Intriguingly, with the assistance of the biodegradable wafer, increasingly biodegradable systems introduced to the parenchyma by surgical implantation have been exploited for localized drug delivery. For instance, based on the template method, the group of Decuzzi shaped poly(lactic‐*co*‐glycolic acid) (PLGA) into micro‐mesh (µMESH) loading with docetaxel and diclofenac for the combinatorial therapy of brain tumors before and after surgical resection.^[^ ^24^ ^]^ Notably, the well‐designed grid structure of this µMESH enhanced the device‐tissue interaction effectively in a flexible and adapted manner, making it more conformable to the complex geometry surface of brain tissue. More encouragingly, biodegradable microneedles also have been introduced into brain tissue as localized drug delivery devices.^[^ ^25^ ^]^ Microneedles pierce into the targeted lesions in a minimally invasive manner, which not only accelerates the deep penetration of the drug, but provides adequate adhesion between devices and parenchyma.^[^ ^26^ ^]^ In addition, the group of Amiji fabricated drug‐loading capsules with a core‐shell structure, which facilitated the implantation surgery with the help of minimally invasive nasal endoscopy instead of traditional craniotomy (Figure 2E).^[^ ^27^ ^]^ Collectively, these localized drug delivery devices introduced by surgical implantation, including wafers, micromesh, microneedles, and microcapsules, are upgrading quickly by pharmacokinetic optimization, geometric adaptation, process simplification, as well as minimally invasive implant routine, to achieve TSPCI.

**Figure 3:**
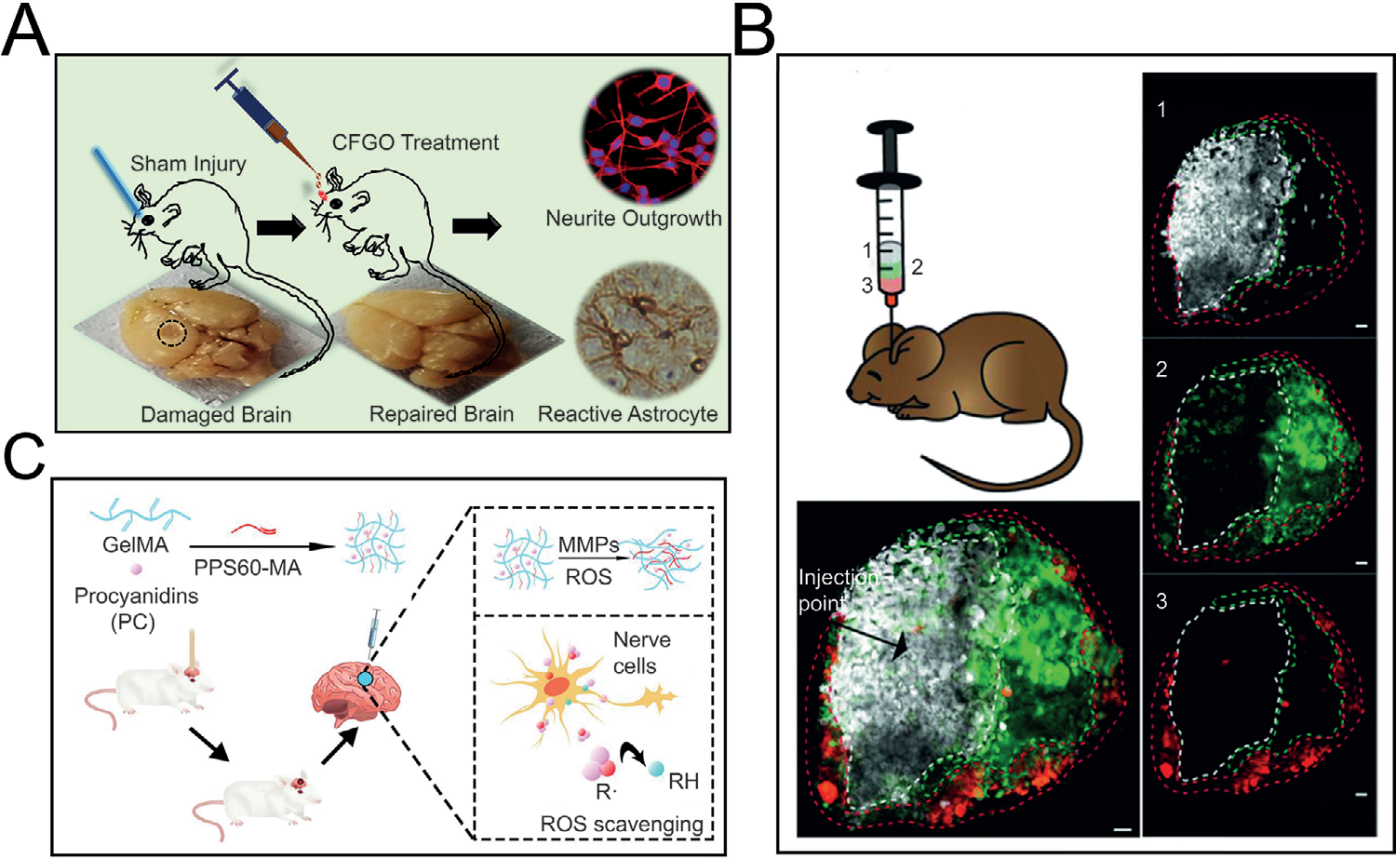
Injectable in situ polymerized hydrogel. A) Acetylcholine‐functionalized graphene oxide (CFGO) based hydrogel for the treatment of brain injury. Reproduced with permission.^[^ ^28^ ^]^ Copyright 2019, American Chemical Society. B) Spatially patterned microporous hydrogels by sequential loading for the treatment of photothrombotic stroke model (scale bar, 100 µm). Reproduced under the terms of the Creative Commons CC‐BY license.^[^ ^29^ ^]^ Copyright 2018, The Authors. Published by Wiley‐VCH Verlag. C) Recombined hydrogel of PPS60 and PC with Gelatin methacrylate (GelMA) as matrix for the treatment of traumatic brain injury. the GelMA‐PPS/PC hydrogel depolymerize in the microenvironment of matrix metalloproteinases (MMPs) and ROS. Reproduced with permission.^[^ ^31^ ^]^ Copyright 2022, American Chemical Society.

Notably, besides surgical implantation, the clinical application of stereotaxic apparatus makes it feasible for precise injection to specific brain regions. Among them, it is the injectable in situ polymerized hydrogel that has been credited as a typical representative. Different from intraventricular injection of drug liquid which not only leads to the diffusion in cerebrospinal fluid, but imposes the potential risks of intracranial pressure increment, injectable hydrogel is blessed with appropriate rheological property for injection, which subsequently undergoes cross‐linking reaction in situ. This solution‐gel transition remarkably retards diffusion of the drug solution, ensuring the precise drug delivery on localized parenchyma. For instance, the group of Ghosh fabricated an acetylcholine‐functionalized hydrogel based on non‐cytotoxic graphene oxide and poly(acrylic acid) to promote neuro‐regenerative and brain injury recovery (**Figure** **3**A).^[^ ^28^ ^]^ More encouragingly, in recent years, spatially patterned injectable hydrogel is gradually established as a mature technique. It is competent for producing arbitrary geometric configuration by subsequential loading and programmed injection without complicated post‐fabrication, therefore endowing the hydrogel with flexible capability of fitting the irregular lesions such as post‐surgery and stroke (Figure 3B).^[^ ^29^ ^]^ Besides spatial precision, intelligent hydrogel manifests distinguished competence in response to various exogenous stimulation (e.g., electricity, thermal, optical wave) and endogenous microenvironment (e.g., reactive oxide species (ROS), pH), paving the way for personalized on‐demand drug therapy.^[^ ^30^ ^]^ For instance, to treat traumatic brain injury, the group of Chen designed a recombined hydrogel of poly(propylene sulfide)60 (PPS60) and procyanidins (PCs), with the former functioning as an activator to trigger the hydrogel depolymerization by responding to H_2_O_2_, and the latter getting rid of ROS to inhibit the secondary injury caused by oxidative stress of damaged brain tissue (Figure 3C).^[^ ^31^ ^]^ In general, in situ polymerized hydrogel possesses comprehensive superiorities of shape‐variability, multifunctionality as well as stimuli‐responsiveness, and is regarded as ideal candidates for TSPCI of NDs.

#### 2.1.2 Non‐Biodegradable (Removable) Systems

Despite numerous merits, biodegradable systems fail in repeated drug loading. removable systems overcome this drawback by establishing temporary catheters for localized parenchyma drug delivery. Convection‐enhancement delivery (CED), a pressure gradient mediated strategy for continuous drug delivery by intracranial catheters, has been developed in enormous preclinical studies and clinical trials in the past decades as adjuvant chemotherapy against diffuse glioma.^[^ ^32^ ^]^ CED is a potential technique to bypass the BBB and synchronously avoid the general toxicity, yet now the long‐term therapeutic effects are uncertain on account of the fast elimination rate of the drug as well as the potential risks of encephaledema induced by the high pressure. Also, the large fluidic outlet size sacrifices the spatial accuracy, and more seriously, aggravates the intracranial infection.

**Figure 4:**
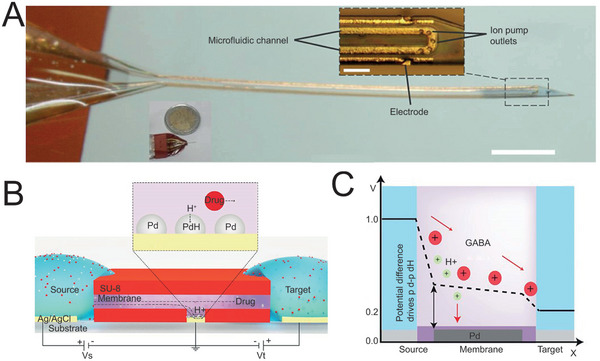
Removeable systems for localized drug delivery. A) implantable‐removable electrode integrated with µFIP to deliver GABA for seizure control (inset scale bar, 100 µm; outside scale bar, 1 mm). Reproduced under the terms of the Creative Commons CC‐BY license.^[^ ^35^ ^]^ Copyright 2018, The Authors. Published by American Association for the Advancement of Science. B) Schematic illustration of proton traps on the ion pump. The H^+^ was captured by Pd when across the membrane. C) The potential difference of H^+^ and the neurotransmitter GABA (drug) from the source pads to the targets. B,C) Reproduced under the terms of the Creative Commons CC‐BY license.^[^ ^37^ ^]^ Copyright 2021, The Authors. Published by American Association for the Advancement of Science.

Another emerging candidate for removable systems is the neural probe. This neoteric localized administration routine has improved the spatial accuracy as well as dosage precision to a new level via the integration with advanced microfluidic and ion pump technologies. Microfluidic technology is qualified for precise manipulation of fluid on the micro‐nano scale, thus providing great opportunities to improve the spatial resolution of drug delivery. For instance, the group of Chiou integrated microfluidic channels into a miniatured multilayer electrode with only 30 µm thickness, which has proven to achieve unparalleled spatial accuracy at single‐cell level.^[^ ^33^ ^]^ Moreover, with the selectively permeable membranes as core components, ion pumps based on the electrophoretic principle could deliver only “dry” drugs without solvent to the lesions, which satisfies the patients’ specific dosage requirement in quantization, and simultaneously avoids cellular edema upon local pressure increment.^[^ ^34^ ^]^ For instance, the group of Malliaras fabricated a neural probe incorporated with a microfluid ion pump (µFIP) to deliver *γ*‐aminobutyric acid (GABA) for seizure control (**Figure** **4**A).^[^ ^35^ ^]^ Connecting microfluidic channels with ion pumps and integrating them with implantable neural probes achieve precise therapeutic control. In addition, besides neural probes, µFIP could also combine with other neural implantable devices. For instance, the group of Williamson and Malliaras integrated µFIP with electrocorticography (ECoG) grids, satisfying the multi‐functionalities of neural recording and precise drug delivery at the cortex.^[^ ^36^ ^]^ Furthermore, the on‐off switch of µFIP could be controlled conveniently by inputting electric current with minimal time delay, consequently providing prerequisites for closed‐loop systems.

Notably, there is an inevitable defect worth mentioning for ion pumps, that under continuous power‐on state, the water solution would be ionized to produce protons which tend to be transported through the anionic membrane along with the drug due to the higher mobility. To overcome this dilemma, the group of Simon introduced proton traps based on palladium (Pd) into the ion exchange membrane.^[^ ^37^ ^]^ Through the electrochemical conversion from Pd to palladium hydride (PdH), protons were absorbed effectively and selectively (Figure 4B,C). Significantly, this clearance fends off the alteration of local pH and damage to healthy brain tissue, improving the clinical transformation potential of ion pump integrated removable systems, particularly for the management of chronic and progressive NDs.

### 2.2 Precise Physical Neuromodulation

Neuromodulation is progressively considered as an essential category for the clinical intervention of NDs. In this section, based on fundamental activation/inhibition principles (non‐genetic and genetics‐based), we systematically investigated the latest progress in physical neuromodulation, with the emphasis on temporal‐spatial precision and clinical transformation potential for TSPCI of NDs.

#### 2.2.1 Non‐Genetic Neuromodulation

Among various physical energy forms, electricity and ultrasound have been confirmed for successful neuron activity modulation directly and safely.^[^ ^38^ ^]^ While the convenient energy transformation such as electromagnetic induction and optoacoustic effect, with/without the assistance of nanophase materials, has significantly enriched the strategies for non‐genetic neuromodulation. Moreover, according to the intervention ways, non‐genetic neuromodulation could be classified into transcranial stimulation and deep brain stimulation (DBS), with the former utilizing electricity, magnetism, or ultrasound and typically targeting at the cortex, while the latter is qualified for targeting specific nucleus in deep brain tissue by implantable stimulators.^[^ ^39^ ^]^

**Figure 5:**
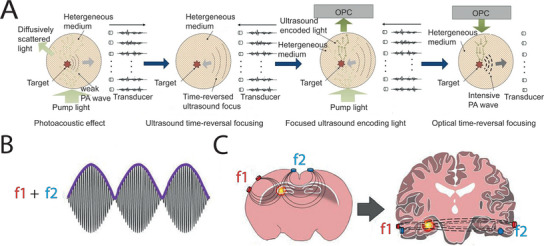
Precise non‐genetic neuromodulation. A) Schematic illustration for photoacoustic (PA) wave time reversal technique. First, a pump light was injected to generate weak PA wave through photoacoustic effect. Subsequently, time reversal was performed on the received PA signal. Then, pump light was injected again to obtained focused ultrasound encoding light, which was detected by optical phase conjugation (OPC). Finally, time reversal was performed on the received optical wave to produce intensive PA. Reproduced with permission.^[^ ^45^ ^]^ Copyright 2022, Elsevier GmbH. B) TI produced by double sine waves (f1: 1300 Hz; f2: 1430 Hz). C) TI stimulation precisely focused on the hippocampus to suppress seizure, without activating the cortex neurons. B,C) Reproduced under the terms of the Creative Commons CC‐BY license. Copyright, 2022, The Authors. Published by Frontiers.

For transcranial stimulation, the spatial orientation of stimulators is the first and foremost issue for the target effects. Unlike the previous strategies relying on blurred scalp markers, nowadays advanced brain imaging technologies such as functional magnetic resonance imaging (fMRI) and positron emission tomography (PET) are accessible to maximize the accuracy and precision of the placement of the coils.^[^ ^40^ ^]^ However, considering the serious and inhomogeneous attenuation effects of the skull, precise orientation of stimulator is not enough to ensure the spatial accuracy.^[^ ^41^ ^]^ Taking ultrasound as an instance, it is an emerging candidate for non‐invasive transcranial neuromodulation with compelling advantages of high spatial resolution, deep tissue penetrability, and excellent biosafety.^[^ ^42^ ^]^ Unfortunately, ultrasonic wave emitted by traditional phased array suffers from focus shift and scattering when penetrating the skull, simultaneously imposing a potential risk of ambustion to the surrounding tissue, hence impeding its clinical applications.^[^ ^43^ ^]^ Facing this challenge, virtual time reversal (VTR) technology is promising to achieve targeted energy focus.^[^ ^44^ ^]^ First, with the assistance of computed tomography (CT) images, the acoustic parameters of the skull are calculated utilizing advanced numerical stimulation without implanting the wave source intracranially (called virtual point), then the analog signals from each array element are reversed into transmitted signals to achieve target tissue. More encouragingly, the group of Wang expanded the cutting‐edge VTR to the field of optical wave by replacing the virtual point with photoacoustic wave, accomplishing the precise focus of optical energy in deep brain tissues (**Figure** **5**A).^[^ ^45^ ^]^ VTR technology is a significant step forward to focus energy on brain lesions by transcranial routine with satisfactory temporal‐spatial precision.

As for DBS, intracranial electrodes are traditionally necessary through surgical operation, during which neuronavigation and intraoperative electrophysiological monitoring play vital roles in guaranteeing the precise spatial orientation of stimulators. However, this invasive manner could asset high demands to surgeons, and may seriously compromise patients’ compliance, thus impeding its extensive clinical application. Recently, temporal interference (TI) is one of the most inspiring technologies to achieve precise and non‐invasive DBS: Single high‐frequency electric field (e.g., >1 kHz) performs better in tissue penetration but fails in neuron activation owing to the intrinsic properties of neural membrane.^[^ ^46^ ^]^ However, when double high‐frequency electric fields with slight difference in frequencies are applied simultaneously on the skull surface, their superimposition could produce a low‐frequency electric field which is capable of neuron activation.^[^ ^47^ ^]^ This cutting‐edge technology makes it possible to perform DBS by non‐invasive (transcranial) routine, achieving decent precision without disturbing the superior cortical neurons. For instance, the group of Williamson reported that TI stimulation with envelop frequency around 130 Hz effectively suppressed the epileptic seizure in the hippocampus of rat models, while transcranial current stimulation in the same frequency failed to reach such deep brain tissue (Figure 5B,C).^[^ ^48^ ^]^ Furthermore, the group of Williamson was devoted to combining TI technology with organic electrolytic photocapacitors which are qualified for laser‐electrical impulse conversion.^[^ ^49^ ^]^ Notably, this laser‐driven wireless TI device liberates the patients from the wired electrodes, which could provide significant convenience for future clinical applications of TI‐based DBS.

#### 2.2.2 Genetics‐Based Neuromodulation

Optogenetics has proven to achieve precise neuromodulation at a single‐neuron level within millisecond, and is undoubtedly a milestone for neuroscience. The following discovery and structural analysis of temperature‐sensitive transient receptor potential (TRP) channel,^[^ ^50^ ^]^ magnetic receptor protein (e.g., iron‐sulfur cluster assembly protein 1^[^ ^51^ ^]^), mechanosensitive ion channel,^[^ ^52^ ^]^ support the further developments of genetics‐based neuromodulation. Importantly, accumulative evidences about the biosafety of adeno‐associated virus (AAV)^[^ ^53^ ^]^ are accelerating its clinical application. Thereinto, essential factors for temporal‐spatial precision and clinical transformation of genetic‐base neuromodulation are illustrated from: 1) stimulation module; 2) genetics‐based receptor protein.

**Figure 6:**
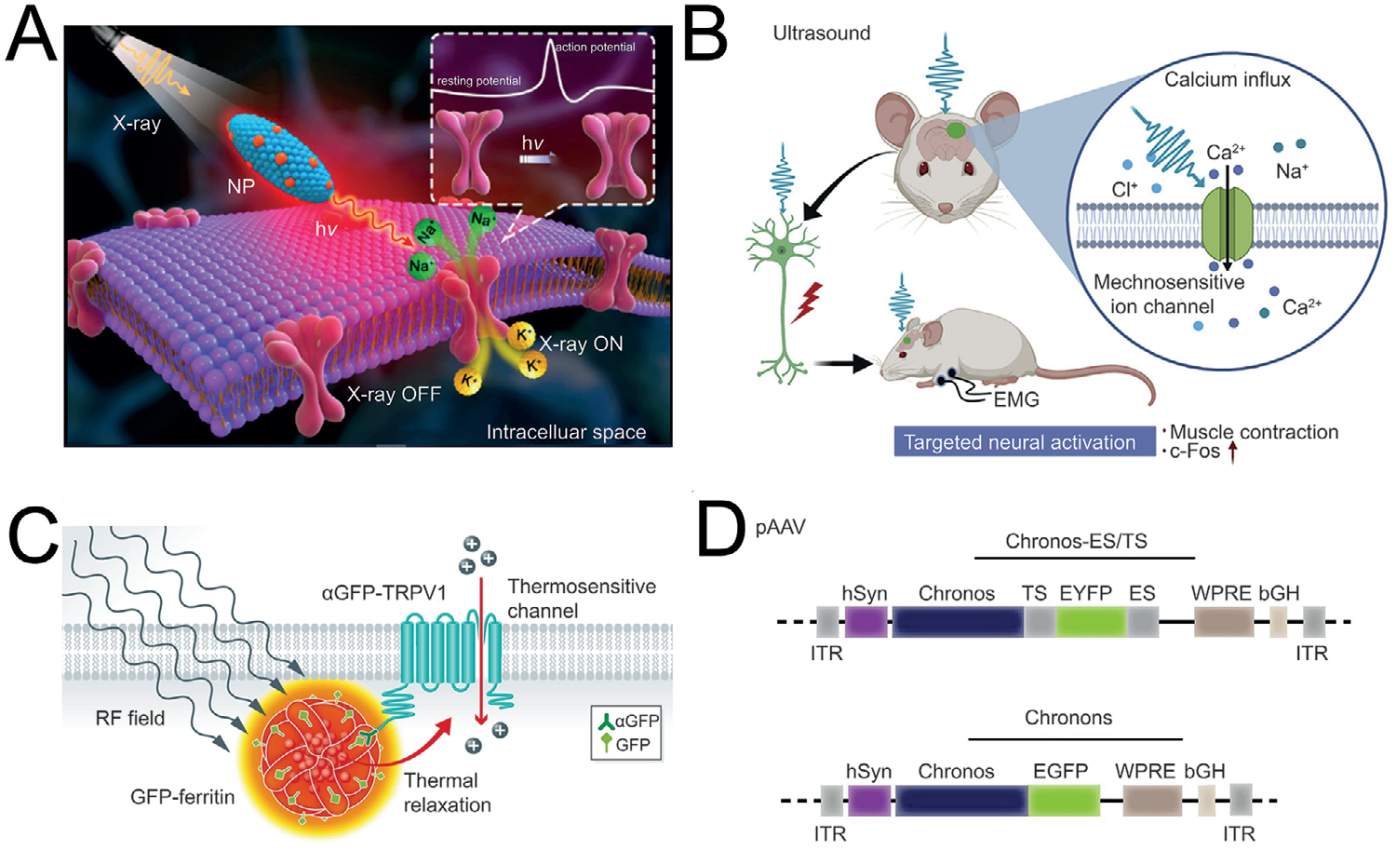
Genetics‐based neuromodulation. A) X‐ray activates ChRs with the assistance of radioluminescent nanoparticles (NP). Reproduced with permission.^[^ ^55^ ^]^ Copyright 2021, American Chemical Society. B) Schematic illustration for the ultrasound to activate mechanosensitive ion channel (MscL‐G22s) in the motor cortex of mice. Electromyogram (EMG) recorded the muscle contraction and the rise of c‐Fos indicated the targeted activation of neurons. Reproduced with permission.^[^ ^56^ ^]^ Copyright 2020, Cell Press. C) Schematic illustration for genetics‐based modification of the magnetothermal nanoparticles with GFP‐ferritin and with TRPV1 anti‐GFP antibody (*α*GFP). Reproduced under the terms of the Creative Commons CC‐BY license.^[^ ^57b^ ^]^ Copyright 2017, The Authors. Published by John Wiley and Sons. D) The genetic sequence of fast Chronos (bottom) and Chronos with adding sequences (top) including export signal (ES) and trafficking signal (TS). Reproduced with permission.^[^ ^58^ ^]^ Copyright 2018, John Wiley and Sons.

For inchoate optogenetics, connected optical fiber or implantable light‐emitting diode (LED) array is integral to produce visible light in certain wavelength, which impedes the clinical utilization, and synchronously compromises the spatial resolution due to the scattering and poor focusing performance of visible light.^[^ ^54^ ^]^ Intriguingly, ingenious nanoparticles capable of yielding visible light by energy transformation from others such as Near Infrared Ray (NIR), X‐ray, have been proposed in recent years. For instance, the group of Rozhkova fabricated radioluminescent nanoparticles which employed Gd_2_(WO_4_)_3_ to absorb X‐ray energy and lanthanide ions (Eu^3+^) to emit light with a maximum wavelength of 610 nm (**Figure** **6**A).^[^ ^55^ ^]^ Combined with red‐shift variant channelrhodopsins (ChRs), the motor cortex of rodent was activated precisely by non‐invasive transcranial route. Similar to optogenetics, the group of Sun reported that transcranial focused ultrasound was qualified for activating specific mechanosensitive channel (Figure 6B).^[^ ^56^ ^]^ Notably, energy forms such as X‐ray and ultrasound are featured in decent skull penetrativity and satisfactory targeting performance, therefore providing opportunity for precise genetics‐based neuromodulation in deep brain tissue non‐invasively.

In addition to the stimulation module, the modification of receptor protein through genetic engineering is another tactic for further improvements. For instance, the group of Friedman modified magnetothermal nanoparticles with green fluorescent protein (GFP), and simultaneously expressed anti‐GFP antibody as tag fusion protein tethered to TRP vanilloid 1 (TRPV1) (Figure 6C).^[^ ^57^ ^]^ The specific antigen‐antibody binding actualized the exact match of generated heat with target neurons, which was indeed an inspirational strategy to enhance the spatial resolution of genetic‐based neuromodulation. In addition, renovation of receptor protein makes it more feasible to satisfy the special application scenarios of NDs. For instance, although the activation for most ChRs is swift, it takes several milliseconds to close, which is unsuitable for auditory sense rescue in high frequency. To solve this contradiction, the group of Moser introduced fast ChRs (Chronos) which spent only sub‐millisecond shutting down after light‐off to spiral ganglion neurons, and concurrently added specific sequences to enhance the membrane expression of Chronos, finally achieving more than 60% spike probability in 100 Hz stimulation frequency (Figure 6D).^[^ ^58^ ^]^

In summary, both stimulation module and receptor protein are indispensable for the temporal‐spatial precision enhancement of genetics‐based neuromodulation. All these inspiring innovations exhibit valuable clinical transformation potential for TSPCI of NDs.

### 2.3 Trigger Factors for Closed‐Loop Control

In physiological/pathological processes, the activities of neurons generate abundant biological signals. From small to large scales, electrical signals of neurons encompass action potential (AP), local field potential (LFP), cortical electroencephalogram (ECoG), and scalp electroencephalogram (scalp EEG).^[^ ^59^ ^]^ Additionally, numerous NDs, especially dyskinesia and neurodegenerative disease, have inextricable associations with the abnormity of specific chemical biomarkers such as neurotransmitters, hormones, metabolites, and so on.^[^ ^60^ ^]^ All these biological signals are valuable in terms of being detected as trigger factors for localized parenchyma drug delivery and precise physical neuromodulation, to achieve real‐time closed‐loop control systems which are qualified for implementing the appropriate intervention timely, and satisfying the personalized requirements precisely (**Table** **1**). In this case, state‐of‐art signal detection techniques have been applied to satisfy superior sensitivity, adequate spatial resolution and minimal time delay. Herein, according to whether the detected signals could be transformed into analog signals or digital signals which are transmissible, the responsive systems are classified into transmissible and transmission‐free types, and we demonstrate the advanced principles and strategies about how biological signals are being detected as trigger factors, as well as the methodologies for signal transmission and TSPCI activation, which are significant for closed‐loop TSPCI of NDs.

**Table 1:** Typical biological signal detection as trigger factors for closed‐loop TSPCI

Device	Signal	Brain region	Signal type	Application	Ref.
Electrode	Electricity	Temporal lobe	Transmissible	Epilepsy	[61]
Electrode (array)	Electricity	Motor cortex	Transmissible	Brain–machine interface	[62]
Electrode (array)	Dopamine	Basal ganglion	Transmissible	Parkinson's disease	[63]
Electrode	Electricity and glutamate	Hippocampus	Transmissible	Epilepsy	[64]
Electrode (array)	Electricity and dopamine	Basal ganglion	Transmissible	Parkinson's disease	[65]
Field‐effect transistor (solution gate)	Electricity	Cortex	Transmissible	Epilepsy	[66]
Field‐effect transistor (solution gate)	Electricity	Cortex	Transmissible	Depression	[67]
Field‐effect transistor (aptamer)	Serotonin	Corpus striatum	Transmissible	Depression	[68]
Field‐effect transistor (aptamer)	Amyloid‐beta peptide (A*β*)	Cortex and hippocampus	Transmissible	Alzheimer's Disease	[69]
Nanoparticles	Electricity	Epilepsy lesions	Transmission‐free	Epilepsy	[70]
Nanoparticles (biomimetic ion channel)	Potassium ion	Epilepsy lesions	Transmission‐free	Epilepsy	[71]

#### 2.3.1 Transmissible Responsive Systems

In closed‐loop control systems for NDs with transmissible signals, the electrical signals or chemical biomarkers are detected by specific testing devices, and transformed into analog signals (usually refers to electric current), which are subsequently transmitted into control modules for integration and analysis, and finally guide the adjustment of intervention modules. In this section, two types of detection devices are introduced to highlight their superiorities for TSPCI of NDs: 1) electrode; 2) field‐effect transistor.

**Figure 7:**
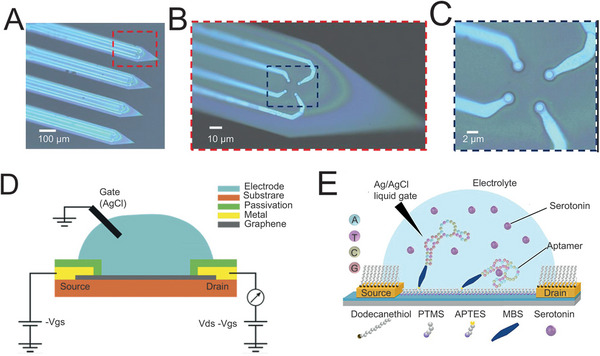
Multichannel electrode array for synergistic detection of electrical and chemical signals. A) Ultramicroelectrode array with four shanks. B) Zoom‐in view of red dashed box in (A), showing four tips on every shank. C) Zoom‐in view of blue dashed box in (B), showing electrode with the diameter of 1 µm on every tip. A‐C) Reproduced with permission.^[^ ^64^ ^]^ Copyright 2020, Elsevier. D) Schematic illustration of solution‐gate FET for the detection of electrical signals of NDs. Reproduced with permission.^[^ ^66^ ^]^ Copyright 2017, John Wiley and Sons. E) Schematic illustration implantable aptamer FET for serotonin detection. PTMS, trimethoxy(propyl)silane; APTES, (3‐aminopropyl)triethoxysilane; MBS, 3‐maleimidobenzoic acid *N*‐hydroxysuccinimide ester. Reproduced under the terms of the Creative Commons CC‐BY license.^[^ ^68^ ^]^ Copyright 2021, The Authors. Published by American Association for the Advancement of Science.

Electrode is unquestionably the most prevailing detection device for both electrical and chemical signals. The challenge lies in the fact that enhancing the spatial resolution by shrinking the volume of tips inevitably increases the impedance between neuron and electrode, which is not conducive to increasing sensitivity and signal‐to‐noise ratio (SNR).^[^ ^72^ ^]^ Tactics to mitigate this contradiction consist of the optimization of electrode material with superior electrical conductivity as well as refined structure design by advanced microfabrication technology.^[^ ^73^ ^]^ Furthermore, integrating dozens or even hundreds of electrodes to form arrays is preferred over a single electrode so as to classify the spike patterns from different neurons.^[^ ^62^, ^63^ ^]^ Importantly, it should be emphasized that a number of NDs integrate the abnormities both in electrical signals and chemical biomarkers. Consequently, multichannel electrodes which are qualified for synergistic detection would enhance the sensitivity and specificity immensely. For instance, the group of Cai fabricated an ultramicroelectrode array which contained four shanks (**Figure** **7**A) with four tips on each of them (Figure 7B) for detecting glutamate (Glu) and electrical signals concurrently to locate epilepsy lesions.^[^ ^64^ ^]^ 16 electrodes with the diameter of 1 µm were distributed orientationally on the tips (Figure 7C), with one of them modified with glutamate oxidase enzyme for Glu concentration monitoring while the others for electrical signals detection. Similarly, the group of Cai reported a microelectrode array with 9 channels for synergistic detection of dopamine and electrical signals in the rat model of Parkinson's disease.^[^ ^65^ ^]^

Another neoteric type of detection device for transmissible signals is field‐effect transistor (FET). Different from the electrode, FET is an active detection device with the capability of signal amplification without extra amplifier module. As shown in Figure 7D, the solution‐gate FET consists of two metallic contacts (namely the source and drain) and a reference electrode (typically Ag/AgCl) immersed into the electrolyte as the gate terminal. Briefly, the change of detected voltage between source and gate (*V*_ds_) results in the resistance variation between source and drain, and when the external source‐drain voltage (*V*_gs_) inputs, the source‐drain current (*I*_ds_) is monitored as a reflection of *V*_ds_.^[^ ^66^, ^67^ ^]^ As the electrical signals of neurons are so faint, FET demonstrates unique superiority in detecting the transmissible signals with high fidelity for further analysis. More inspiringly, when coupled with aptamers, FET is endowed with the capability for monitoring the concentration of specific neurotransmitters precisely. For instance, the group of Andrews developed an implantable FET for the detection of serotonin by modifying the surface of In_2_O_3_ with synthetic oligonucleotide receptors, indicating the detection limit at femtomolar level (Figure 7E).^[^ ^68^ ^]^ Importantly, the application of this strategy could be extended to other neurotransmitters flexibly by the alternation of specific aptamers.^[^ ^74^ ^]^ Featured in multifunctionality, flexibility, satisfying sensitivity, and miniature size, FET exhibits great value for TSCPI of NDs.

Collectively, these advanced detection devices for transmissible signals are set as fundamental for closed‐loop TSPCI of NDs. The following signal transmission processes depend on wired/wireless transmission channels. For instance, optical fibers combined with microelectrodes allow for electrical/chemical signal transmission to control modules, and meanwhile the reversed optical waves to the brain tissue for genetic‐based neuromodulation, achieving timely and precise closed‐loop control.^[^ ^75^ ^]^ While in comparison, wireless transmission systems would be preferred for clinical application, owing to their advantages including miniatured size, light weight, mobility, portability, and personal adaptation.^[^ ^76^ ^]^ Currently, diverse wireless devices are commercially available for detected signal transmission to control modules (e.g., mobile phones, wearable watches) after suitable transformation into digital/analog signals. Encouragingly, drug release and neuromodulation activation could be accomplished by wireless energy forms such as X‐ray, ultrasound, therefore creating promising strategies for wirelessly closed‐loop control.^[^ ^42^, ^55^ ^]^ For instance, the group of Kim fabricated a soft implantable anti‐epileptic drug delivery device, which could be timely triggered by wireless radio frequency signal once the control modules received alarms form another wearable electroencephalography signal monitoring device.^[^ ^77^ ^]^ Notably, this innovation has proposed a significant strategy for the first‐aid of fatal seizure. Moreover, transmissible signal responsive systems for closed‐loop TSPCI are also inseparable with intellective algorithm for signal postprocessing and integration so as to guide the adjustment of output parameters. This is challenging owing to the intricate pathophysiology of NDs. Nevertheless, combined with contemporary artificial intelligence technologies, it is a promising trend to achieve the closed‐loop TSPCI.^[^ ^78^ ^]^ Specifically, single‐parameter model should be transitioned to multiple‐parameter integration for both input and output, to better adapt to the dynamic patient's conditions.

#### 2.3.2 Transmission‐Free Responsive Systems

Giving credit to cutting‐edge chemical engineering techniques, transmission‐free responsive systems have been explored in recent years for closed‐loop TSPCI of NDs. Based on specific reaction mechanisms, certain materials go through phase transition or depolymerization in response to biological signals of NDs and release the loading drugs in lesions timely and precisely. These concise systems accomplish closed‐loop intervention free from complex signal transformation and extra control modules, indicating the valuable potential for clinical applications. In this section, detailed principles and strategies are illustrated from two aspects according to the original type of detected signals: 1) electrical signal; 2) chemical biomarker.

**Figure 8:**
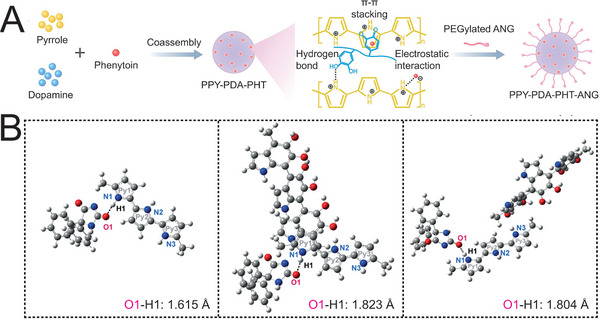
Non‐transmissible electrical signal‐responsive systems. A) Schematic illustration of the synthesis of electro‐responsive nanoparticles loading with phenytoin for seizure control. Angiopep‐2 (ANG), a 19‐mer peptide, was introduced to enhance the BBB penetration. B) Schematic illustration of the interaction between PHT molecule and PPY polymer chain without PDA (left), with PDA bonded to the second PPY molecule (middle), and with PDA bonded to the third PPY molecule (right). The introduction of PDA extends the bond length of O—H between PHT and the first PPY molecule, decreasing the bond energy. A,B) Reproduced under the terms of the Creative Commons CC‐BY license.^[^ ^70^ ^]^ Copyright 2022, The Authors. Published by American Association for the Advancement of Science.

As numerous NDs are characterized with abnormal electrical activities of neurons, the responsive drug release mechanisms with electric stimulus are crucial for on‐demand intervention. Based on the ionic current flow during epileptic seizure and the osmotic pressure variation of lesions, the group of Ying developed electro‐responsive hydrogel which could be deformed and release phenytoin (PHT) with superb spatial precision,^[^ ^79^ ^]^ while further enhancements are expected in response speed and sensitivity. Actually, for compounds rich in conjugated systems, the depolymerization could be induced directly by electric stimulus owing to the migration and hopping of electrons. Typical prototypes contain thin films based on layer by layer self‐assembly, as well as nanoparticles integration by chemical copolymerization.^[^ ^80^ ^]^ For instance, Wang and coworkers fabricated electro‐responsive nanoparticles by pyrrole (PPY) and polydopamine (PDA), in which the incorporation of PDA at a ratio of 5% led to 125‐fold increment of electrical conductivity compared with that of pure PPY (**Figure** **8**A).^[^ ^70^ ^]^ The underlying mechanism is probably that the interaction between PDA and PPY decreased the intermolecular bond energy of PPY and PHT, according to density functional theory (Figure 8B).^[^ ^81^ ^]^ Encouragingly, these explorations provide novel insights for closed‐loop control of epilepsy as well as other NDs with abnormal electrical signals.

**Figure 9:**
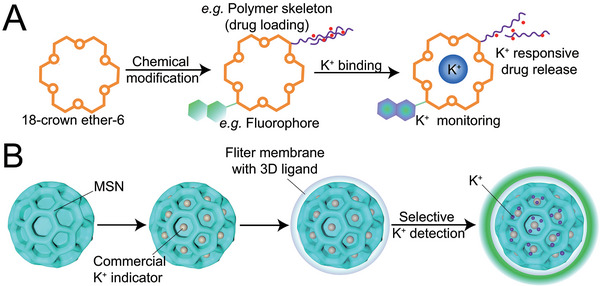
Non‐transmissible K^+^ responsive systems A) 18‐crown ether‐6 possessed a big ring structure for selective K^+^ binding. With suitable chemical modification (e.g., fluorophore, polymer skeleton), it achieved K^+^ concentration monitoring and responsive drug release. B) Commercial K^+^ was loaded on MSN. When coated with a filter membrane with special 3D ligand, K^+^ was selectively detected while Na ^+^ was excluded.

Additionally, there are specific natural or synthetic compounds possessing suitable selectivity to specific chemical biomarkers, inorganic ions in especial, thus providing another manner for non‐transmissible signal detection systems to achieve closed‐loop control. For instance, alternation of potassium ions (K^+^) level has been proven to associate with NDs including epilepsy and AD.^[^ ^82^ ^]^ Based on this phenomenon, early attempts relative to potassium ion‐responsive drug release systems focused on chemical compounds such as 18‐crown ether‐6, valinomycin, as they shared similar molecular structures with big rings suitable for selective hydrated potassium ions binding (**Figure** **9**A).^[^ ^83^ ^]^ However, it should be noted that toxicity and poor biocompatibility hamper their clinical transformation tremendously. Inspiringly, Ling and coworkers reported a nanosensor based on mesoporous silica nanoparticles (MSNs) loaded with a commercial indicator for potassium ion detection (Figure 9B).^[^ ^71^ ^]^ As the indicator failed in distinguishing sodium ion (Na^+^) from K^+^, while the diameters of both hydrated Na^+^ (7.16 Å) and hydrated K^+^ (6.62 Å) are much smaller than the size of MSNs (3 mm), filter membrane with 3D ligand was designed to shield the nanosensor, avoiding the interference of Na^+^. The cavity radium formed by the ligand was 2.85 Å, very close to the radium of K^+^ without hydration. Consequently, when K^+^ was located near the nanosensor, K—O bond was formed to help K^+^ shed the hydration shell, and finally enter the filter membrane. Similar procedure failed to occur for Na^+^ owing to the mismatch between its radium to that of the cavity, resulting in Na^+^ exclusion. Interestingly, this mechanism upon selectivity between K^+^ and Na^+^ is quite similar to that of K^+^ channel protein, simultaneously satisfying admirable sensitivity and non‐invasiveness.^[^ ^84^ ^]^ Importantly, when combined with drug delivery, this innovation demonstrates valuable clinical potential for on‐demand intervention of NDs characterized with abnormal K^+^ level.

To sum up, despite the initial stage, the superiority of transmission‐free responsive systems has attracted increasing interests of researchers. With further development, it is predictable that these closed‐loop control systems would be expanded to other NDs, turning into an essential subcategory for TSPCI of NDs.

## 3 Promising Clinical Applications of TSPCI for NDs

NDs contain diverse diseases resulting from the dysfunction of neural circuits, whether for central nervous system (CNS) or peripheral nervous system (PNS), almost without exception calling for TSPCI. It should be noted that the goal of developing plentiful advanced strategies of TSPCI is to satisfy clinical treatment requirements. In the past decades, several TSPCI strategies have been approved by FDA for glioblastoma after surgery, drug non‐responsive epilepsy, and so on (**Table** **2**). However, further improvements are essential to enhance curative effects as well as expand the indications. In this section, we chose specific NDs (intracranial tumors, focal epilepsy, essential tremor, ventricular tachycardia) as typical instances to illustrate the promising clinical applications of the TSPCI.

**Table 2:** Typical FDA‐approved TSPCI of NDs

Intervention strategy	Product name	Company	Application	Premarket approval (PMA)	Year
Electrical neuromodulation (spine)	eCoin peripheral neurostimulator	Valencia Technologies Corporation	Urinary incontinence	P200036	2022
Electrical neuromodulation (spine)	Senza spinal cord stimulation (SCS) system	Nevro Corporation	Chronic pain	P130022/S042	2022
Electrical stimulation (ventral intermediate nucleus)	Vercise PC, Vercise Grevia, and Vercise Genus deep brain stimulation (DBS) Systems	Boston Scientific Corporation	Essential tremor/Parkinsonian tremor	P150031/S040	2021
Electrical neuromodulation (brain)	Abbott InfinityTM DBS neurostimulation system	Abbott Medical	Parkinson's disease	P140009/S039	2020
Electrical neuromodulation (brain)	Inspire upper airway stimulation (UAS)	Inspire Medical Systems, Inc.	Obstructive sleep apnea	P130008/S039	2020
Electrical stimulation (sacral nerve)	Axonics sacral neuromodulation system	Axonics Modulation Technologies, Inc.	Urinary incontinence	P190006	2019

### 3.1 Intracranial Tumors

**Figure 10:**
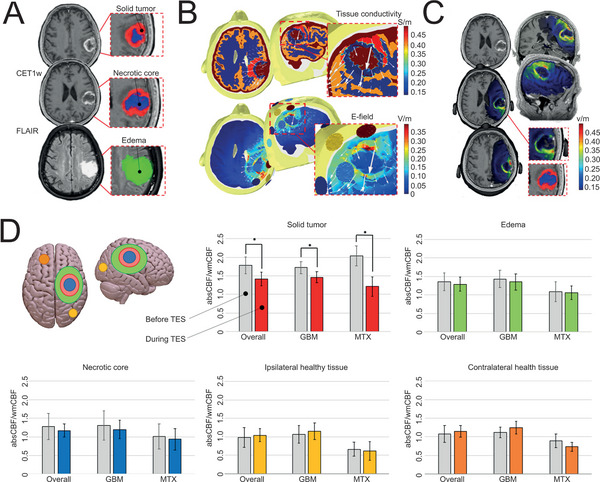
Non‐invasive TES for the treatment of glioblastoma. A) MRI images including contrast‐enhanced T1w (CET1w) and fluid‐attenuated inversion recovery (FLAIR) were applied to each patient, following with manual segmentation to sort out solid part of tumor (red), necrotic core (blue) and edema (green). B) Conductivity values and E‐field distribution were assigned on patients’ head models. C) E‐field results were overlaid onto individual anatomical T1w scans, showing the specificity of the TES solution targeting the solid tumor. D) CBF images before and during TES. The absolute CBF (absCBF) was corrected for CBF of contralateral health white matter (wmCBF, normal CBF). A‐D) Reproduced under the terms of the Creative Commons CC‐BY license.^[^ ^88^ ^]^ Copyright 2019, The Authors. Published by American Association for the Advancement of Science.

Intracranial tumors include primary tumors (e.g., glioma, originating from neuroglial cells) and brain metastases (MTX), with representative clinical manifestations of cerebral dysfunction, cognitive deficit, and epileptic seizure ascribing to perineural invasion.^[^ ^85^ ^]^ Localized parenchyma drug delivery strategies including biodegradable systems as well as CED are promising to avoid the intolerable systemic toxicity of chemotherapy, and rescue the neurological function, achieving effective TSPCI against intracranial tumors.^[^ ^20^, ^32^, ^86^ ^]^ Moreover, besides chemotherapy, sonodynamic therapy based on ultrasound related to sonosensitizers such as 5‐aminolevulinic acid, is one of the promising comprehensive therapies for intracranial tumors, owing to the superior penetrating and focusing properties of ultrasound compared with that of light for photodynamic therapy.^[^ ^87^ ^]^ Intriguingly, bleeding‐edge researches reveal that brain stimulation (neuromodulation) technologies are likely to be an augmentative strategy for retarding tumor growth. For instance, the group of Santarnecchi conducted a single‐center clinical trial to investigate the therapeutic effects of transcranial electrical stimulation (TES) for glioblastoma (GBM) and brain MTX.^[^ ^88^ ^]^ Personalized TES was carefully designed with the guide of neuroimaging technologies as well as the tissue conductivity analysis to ensure that the electrical field was located precisely within the solid tumor (**Figure** **10**A–C). Encouragingly, cerebral blood flow (CBF) images indicated that through TES treatment, the blood flow for both solid tumor and necrotic core were reduced, without edema enlargement or healthy brain tissue damage (Figure 10D). These evidences verified that TES was capable of inhibiting the angiogenesis and tumor metastasis. Probably, electrical stimulation interferes the contact between the tumor cells and healthy neurons, and simultaneously destructs the microenvironment for tumor growth.^[^ ^89^ ^]^ Moreover, the oscillation and mechanical interference induced by low‐intensity alternating magnetic fields might retard tumor progression, based on underlying mechanisms including intracellular calcium overload, oxidative stress, autophagy, and the programmed death of malignant cells.^[^ ^90^ ^]^ Although further studies are necessary to elucidate detailed mechanisms and explore the effectiveness of other brain stimulation strategies, these discoveries have provided new insights for TSPCI of intracranial tumors.

### 3.2 Focal Epilepsy

**Figure 11:**
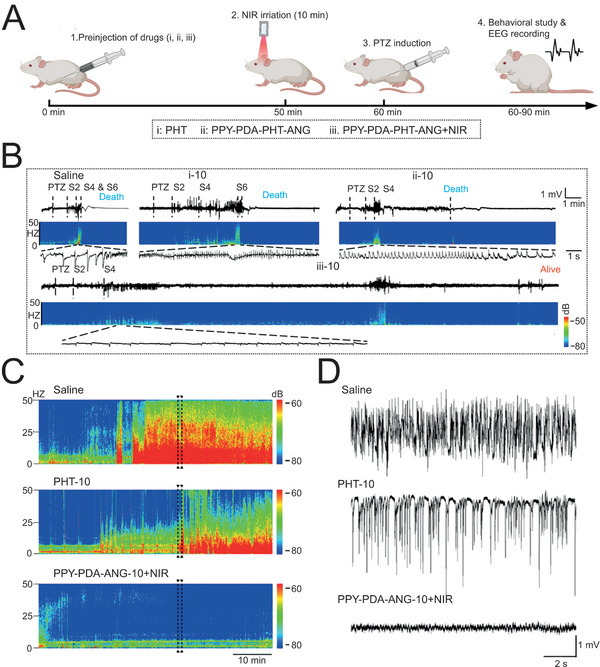
Electric responsive nanoparticles for on‐demand drug delivery. A) Schematic illustration of treating acute seizure model induced by PTZ. B) EEGs and corresponding power spectral analysis at the primary motor cortex after different treatments at a dose of 10 mg kg^−1^ PHT. C,D) Power spectral analysis and representative EEGs at hippocampus of SE model induced by pilocarpine with different treatments at a dose of 10 mg kg^−1^ PHT. A‐D) Reproduced under the terms of the Creative Commons CC‐BY license.^[^ ^70^ ^]^ Copyright 2022, The Authors. Published by American Association for the Advancement of Science.

Focal epilepsy (FE) has confined seizure focus but potentially turns into grand mal epilepsy without timely and appropriate intervention. Consequently, closed‐loop control systems are particularly momentous for FE. Responsive neurostimulation system (RNS) is the first implantable closed‐loop device approved by FDA in 2013 for adults with drug‐resistant FE.^[^ ^91^ ^]^ Notably, though over 70% of FE could be controlled with drugs, long‐term administration is obligatory to ensure effective plasma concentrations, which could exert side effects including nausea, vomiting, headache, and somnolence.^[^ ^92^ ^]^ In this case, it is significant to exploit on‐demand drug delivery systems to improve current treatments. Wang and coworkers fabricated the electro‐responsive nanoparticles loaded with PHT for timely control of seizure, with the modification of Angiopep‐2 (ANG) and the assistance of NIR to enhance brain‐target effects.^[^ ^70^ ^]^ Inspiringly, in rodent models of acute seizure induced by pentylenetetrazole (PTZ), the preinjection of fabricated nanoparticles effectively reduced the severity of the seizure and prolonged survival at the dose of 10 mg kg^−1^ PHT (**Figure** **11**A,B). Moreover, the EEG monitor illustrated that the status epilepticus (SE) induced by pilocarpine was blocked remarkably, indicating the validity of this intelligent intervention strategy for various types of seizure (Figure 11C,D). Importantly, drug release was negligible without electric stimulus, efficaciously reducing the drug dose and enhancing the temporal‐spatial precision. Collectively, all these advanced closed‐loop control systems including neuromodulation and drug delivery are believed to bring a new dawn for TSPCI of FE.

### 3.3 Essential Tremor

**Figure 12:**
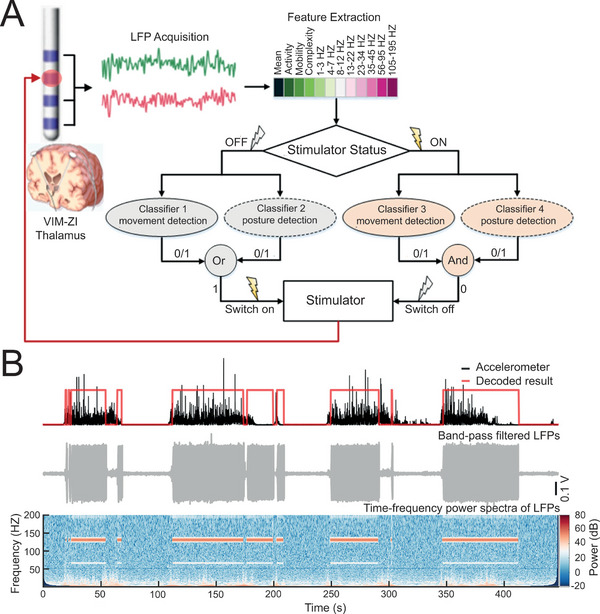
Implantable in situ closed‐loop DBS system for ET treatment. A) The mechanisms and processes for closed‐loop control. B) The matching effect between decoded result (0/1) with physical signals detected by accelerometer (upon), and corresponding filtered LFPs (middle) and time‐frequency power spectra (bottom) detected from VIM and ZI, indicating satisfactory matching‐degree. A,B) Reproduced under the terms of the Creative Commons CC‐BY license.^[^ ^97^ ^]^ Copyright 2021, The Authors. Published by John Wiley and Sons Inc.

Essential tremor (ET) is a common type of movement disorder, with the typical symptom of kinetic tremor, which means that the involuntary tremor aggravates during the process of goal‐targeting, therefore compromising daily activities such as writing, holding objects, and managing tools.^[^ ^93^ ^]^ Without the disease‐specific drug, the existing medication seems to be ineffective for over a half of the patients. Encouragingly, FDA has approved DBS for ET treatment, attenuating the tremor symptoms in over 90% of patients.^[^ ^94^ ^]^ While this open‐loop and constant stimulation is not only energy‐wasting, but prone to induce resistance and undesired adverse effects.^[^ ^95^ ^]^ Intriguingly, based on the invariable association between motor movement and tremor occur, several attempts have been proposed to trigger the closed‐loop stimulation for ET by movement monitoring. For instance, Gunduz and coworkers developed an implantable cortico‐thalamic closed‐loop DBS system, which consisted of subdural electrode arrays at the primary motor cortex for electrical signal detection, followed by computer algorithm analysis to sort out the distribution of power and frequency, and finally actuate the stimulation implanted in ventralis intermediate (VIM) nucleus.^[^ ^96^ ^]^ Notably, on‐demand stimulation was delivered with minimal time delay (with an average of 1.21 s), and matched perfectly with the physiological signals of the contralateral hand according to the accelerometer traces worn on patients’ hands. Furthermore, the group of Tan reported that by extracting features of LFPs from VIM and zona incerta (ZI) at thalamus, it is likewise feasible to trigger on‐demand stimulation for ET (**Figure** **12**A).^[^ ^97^ ^]^ Importantly, this neurotic in situ LFPs detection achieved satisfactory matching‐degree with movement/posture (Figure 12B), paving the way for closed‐loop TSPCI of movement disorders free from separated sensor modules. Notably, besides invasive DBS, thalamotomy based on MRI‐guided focused ultrasound (FUS) is free from risky craniotomy, implantable electrodes, ionizing radiation, and age limitation, thus has provided another decent choice for intractable VT patients.^[^ ^98^ ^]^ Encouragingly, four‐year follow‐up results indicated sustained clinical benefits with rare side effects.^[^ ^99^ ^]^

### 3.4 Ventricular Tachycardia

**Figure 13:**
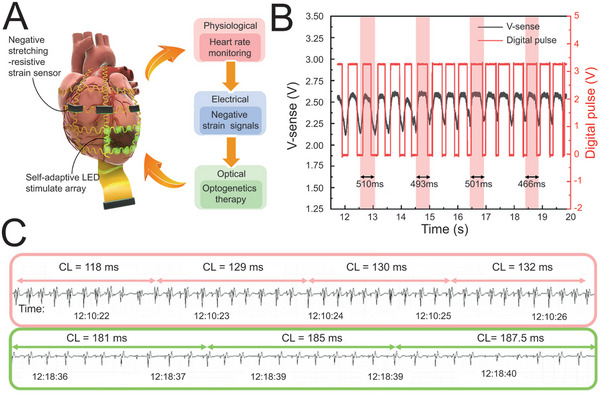
Self‐adaptive system for closed‐loop control of VT. A) Schematic illustration for the component and mechanism of the system. B) Analog strain signal (V‐sense, left axis) obtained from the strain sensor array and the corresponding digital pulses (right axis), showing an estimated heart rate close to 120 beats min^−1^. C) Average CL before (upon) and after (bottom) optogenetics treatment. A‐C) Reproduced under the terms of the Creative Commons CC‐BY license.^[^ ^105^ ^]^ Copyright 2021, The Authors. Published by American Association for the Advancement of Science.

The cardiac conduction systems play a vital role in the normal rhythm of heart beating. Both inherited diseases (e.g., long QT interval syndrome caused by ion channel abnormity^[^ ^100^ ^]^) and acquired factors (e.g., coronary heart disease,^[^ ^101^ ^]^ viral myocarditis^[^ ^102^ ^]^) could cause the damage to the cardiac conduction systems, therefore resulting in various types of undesirable arrhythmia. Among them, ventricular tachycardia (VT) is a common yet could be a fatal type without appropriate and timely intervention. Persistent VT could lead to severe hemodynamic disturbance, even ventricular fibrillation and sudden cardiac death, therefore urgently calling for the precise and closed‐loop control with minimal time delay. In the clinic, cardiac pacemaker implantation has been approved to serve VT beyond the control of drug therapy.^[^ ^103^ ^]^ However, application of high‐energy electric pulse is the prerequisite to depolarize the entire heart before returning to a normal rate, and repeated depolarization may result in the contrary aggravates arrhythmia.^[^ ^104^ ^]^ Intriguingly, optogenetics‐based neuromodulation with advanced temporal and spatial precision has gradually been employed in cardiology. For instance, the group of Liu reported a self‐adaptive system which encompassed a negative stretching‐resistive strain sensor and an optogenetic stimulation module for the closed‐loop intervention of VT (**Figure** **13**A).^[^ ^105^ ^]^ Based on multi‐walled carbon nanotube and natural latex, a special membrane with negative resistance variation of strain during stretching was fabricated, to monitor the contraction and relaxation of heart constantly, and transform the heart rate (physiological signal) into electrical signals (Figure 13B). In addition, LED arrays were anchored into the flexible substrate and controlled by processing circuit, to accomplish optogenetic stimulation once the VT was detected. Impressively, this self‐adaptive system blocked VT effectively and achieved 58.9% increase in cycle length (CL) in 8 min after stimulation (Figure 13C). Notably, it is the precision that is deemed as most effective factor for energy saving, at the meantime playing cardioprotective effects.

Encouragingly, despite at the initial stage, it is an emerging field in recent years to develop closed‐loop TSPCI systems for timely control of other peripheral NDs. For instance, the group of Rogers fabricated an implantable optogenetic modulation device for the closed‐loop treatment of overactive bladder.^[^ ^106^ ^]^ Meanwhile, the group of Sunagawa proposed a neoteric blood pressure control manner by arterial baroreflex stimulation, which in combination with artificial pressure sensor to achieve biomimetic closed‐loop adjustment.^[^ ^107^ ^]^ It is believed that these innovations could exhibit great clinically translational potential for peripheral NDs, expanding the application of TSPCI systems.

## 4 Challenges and Perspectives for Clinical Translation of TSPCI

With temporal‐spatial precision gradually embedded into both doctors and patients’ beliefs, TSPCI demonstrates the promising potential for current and future clinical applications. Recently, a new generation of TSPCI for NDs is under clinical trial, with the exploration in novel intervention strategies, pathways, targets, devices, and indications (**Table** **3**). Yet the way to the clinic/market could be full of challenges relative to acute implantation response, long‐term biosafety concern, device duration, convenience and comfort for utilization, techniques and cost for scaled production, and so forth. In this section, we carefully illustrated the challenges and perspectives for clinical translation of TSPCI, from the aspects of 1) minimal invasive routes for implantation/introduction; 2) long‐term biosafety and device duration; 3) therapeutic effect enhancement and indication expansion; 4) industrialization, to provide guidance for future products and expedite the way to the clinic/market.

**Table 3:** Typical clinical trials of TSPCI for NDs

Intervention	Innovation	Participants	CT phase	Cov identifier	Application	Year
Directional deep brain stimulation	Novel intervention strategy	84	N/A	NCT05418894	Treatment resistant depression and epilepsy	2022
Optogenetic‐based neuromodulation	Novel intervention strategy	6	Phase 2	NCT05417126	Stargardt disease	2022
Transcutaneous vagal nerve stimulation	Novel intervention routine	66	N/A	NCT05180916	Focal epilepsy	2022
Acoustic stimulation	Novel indication	12	N/A	NCT05184270	Parkinson's disease	2022
Deep brain stimulation (medial pulvinar thalamic nucleus)	Novel target	12	N/A	NCT04692701	Epilepsy	2021
Deep brain stimulation (Asymmetric Subthalamic nucleus)	Novel target	12	N/A	NCT03462082	Axial motor dysfunction of Parkinson's disease	2021
Selective left subclavian ansae stimulation	Novel target	30	N/A	NCT05133414	Atrial fibrillation	2021
Transcranial magnetic stimulation	Novel indication	40	N/A	NCT04644614	Urinary incontinence	2020
Transcranial magnetic stimulation	Novel indication	30	Phase 1	NCT04482179	Alzheimer disease	2020
Functional electrical stimulation	Novel indication	40	N/A	NCT04269798	Cerebral palsy	2020
Deep brain stimulation (Abbott Laboratories Infinity implantable system)	Novel device	12	N/A	NCT03952962	Treatment resistant depression	2019
Optogenetic‐based neuromodulation	Novel intervention strategy	15	Phase 1/phase 2	NCT03326336	Non‐syndromic Retinitis Pigmentosa	2017
Convection Enhanced delivery of Nanoliposomal irinotecan (nal‐IRI)	Novel intervention strategy	6	Phase 1	NCT03086616	Diffuse intrinsic Pontine Glioma	2017
Convection enhanced delivery/AAV2‐GDNF	Novel intervention strategy	25	Phase 1	NCT01621581	Parkinson's disease	2012
Neuronavigation and subcortical stimulation	Novel target	58	N/A	NCT01351337	Glioma and Motor Pathway	2011

### 4.1 Minimally Invasive Routes for Implantation/Introduction

Introduce routes are the first issues that should be concerned for TSPCI, especially for localized parenchyma drug delivery, DBS via intracranial neuromodulation, and transmissible responsive systems for closed‐loop control, which rely on intracranial implantation. Despite benefits, the potential risks including infection, hemorrhage, encephaledema, and neuronal function impairment, could dampen patients’ positivity for treatments, and simultaneously pose a burden for medical workers.^[^ ^108^ ^]^ Notably, there are intimate connections between implantation/introduction routes and the periods for safety evaluation before FDA approval. For instance, a neural prosthesis named Stentrode from Synchron company has been approved for clinical trial in 2022 for the treatment of paralysis, racing ahead of congeneric products from Neuralink company, which to a great extent should be ascribed to the fact that instead of traditional craniotomy, vascular intervention through carotid artery facilitates Stentrode implantation in a minimally invasive route. Besides vascular intervention, advanced neuroendoscope and microsurgery techniques have provided bright opportunities for operative route innovations of neuroimplantable devices. Importantly, on this basis, shape memory materials which are competent for compression into miniatured sizes while expansion at targets, will demonstrate unique superiority to minimize the implantation invasiveness. Inspiringly, shape memory alloys (e.g., Ni–Ti alloys) have been widely applied in surgical implants such as endovascular stent,^[^ ^109^ ^]^ articular orthotist,^[^ ^110^ ^]^ and macromolecular hydrogels with elastic polymer network structures (e.g., polyurethane,^[^ ^111^ ^]^ silk fibroin^[^ ^112^ ^]^) have been developed as tissue engineering stents. These clinical practices have provided fundamental insurance about the biocompatibility of specific shape memory materials, indicating promising potential to produce a new generation of neural implants equipped with shape memory capabilities, for localized drug delivery and precise neuromodulation.

Additionally, given the fragility of brain tissue, great emphasis should be placed upon flexibility for material optimization, besides the basic requirement of biocompatibility. While to provisionally enhance the Young's modulus during the implantation processes, the assistance of stiff agents is still indispensable, which may raise the risk of acute implantation response. Therefore, exploiting novel implantation techniques is crucial to mitigate these contradictions. For instance, the group of Robinson reported that tension force generated by a microfluidic device was competent to expedite the implantation of ultraflexible electrodes without stiff agent.^[^ ^113^ ^]^ Moreover, this extracranial “fluidic microdrive” avoided the intracranial pressure alternation and brain tissue damage by redirecting the fluid at the injection point, achieving the introduction of flexible implants with satisfactory accuracy in a minimally invasive manner. Other favorable strategies to alleviate acute implantation response may include utilizing biodegradable assistant agents,^[^ ^114^ ^]^ or coating with anti‐inflammatory and neuroprotective drugs at the surface of implants.^[^ ^115^ ^]^ Collectively, in terms of minimal invasive routes for implantation/introduce, the size of implants, the optimization of materials with flexibly and/or shape memory characteristics, the assistance of advanced neurosurgery techniques, and the innovation of implantation strategies should be taken into consideration carefully for novel TSPCI of NDs, which are beneficial to curtail the development period and cost, as well as improve the comfort and compliance of patients.

### 4.2 Long‐Term Biosafety and Device Duration

After a successful introduction, long‐term biosafety and device duration become critical factors to stabilize the therapeutic effects of TSPCI. Notably, the chronic irritation of foreign substances could result in inflammatory response with the characteristics of glial proliferation and neuron necrosis, which not only is prejudicial to nerve‐device interaction, but more seriously may turn into epilepsy lesions. Moreover, the electronic device duration is heavily restricted by battery capacity and performance attrition of devices, indicating the possibility of secondary operation for replacement. These issues should catch the researchers’ attention at the early stage, to reduce clinical trial failure or market removal.

Surface modification has been proven as an effective and dominant strategy to alleviate chronic inflammatory reactions. Given the unmatched stiffness and mechanical stimulation of bare metal, numerous biomaterials such as polyamide, chitosan, astaxanthin, and neurotic homolog plant protein,^[^ ^116^ ^]^ have been adopted/explored as coating layers. Moreover, introducing anti‐adhesion drugs (e.g., heparin^[^ ^117^ ^]^) to play synergetic effects of gliosis resistance is currently a significant research point of neuropharmacology. While it should be pointed out that the protective validity could be sacrificed once the biomaterials and drugs are metabolized and absorbed, making it questionable for durable services. Fortunately, the cooperation of advanced micromachining techniques (e.g., microimprint lithography^[^ ^118^ ^]^) on metallic surface and surficial deposition methods (e.g., physical/chemical vapor deposition^[^ ^119^ ^]^) for coating materials could remarkably increase their contact areas with uniform sedimentation, which are promising to prolong the life‐spans of novel neural implants. Intriguingly, ongoing researches about cell adhesion behavior have prompted the speculation that these well‐designed nanoengineering structures could modulate the migration and differentiation of gliocyte through the integrin‐mediated mechanosening,^[^ ^120^ ^]^ indicating the inspiring possibility of permanent anti‐gliosis effect.

In terms of reoperation concern for battery replacement, smart wireless charging technology might be an intelligent alternative to the traditional storage battery. Physical energy forms (e.g., ultrasound, magnetic field) which are competent for skull penetration could be collected and transformed into electricity for recharge via piezoelectric effect, and electromagnetic induction principles.^[^ ^121^ ^]^ Specifically, state‐of‐art piezoelectric materials are integrated with the advantages of high energy‐harvest efficiency, well‐adapted geometric configuration, excellent biocompatibility and low cost,^[^ ^122^ ^]^ thus are suitable candidates for wireless recharge of neural implants, especially when combined with precise transcranial focused ultrasound.^[^ ^45^ ^]^ Furthermore, if implantable biofuel cells are adopted, energy resources inside the human body (e.g., glucose^[^ ^123^ ^]^) could be oxidized to provide inexhaustible electricity for neural implants. It is believed that the integration with these cutting‐edge techniques and functional materials would bestow excellent duration upon TSPCI of NDs.

For genetic‐based neuromodulation, however, although it could be free from duration limitation with the assistance of wireless transcranial actuation (e.g., X‐ray,^[^ ^55^ ^]^ ultrasound,^[^ ^56^ ^]^ magnetic field^[^ ^57a^ ^]^), as an irreversible intervention strategy for neural systems, more rigorous preclinical/clinical researches might be required to investigate its long‐term biosafety. While optimistically speaking, with the accumulation of clinical evidences about targeting effects and security of AAV, genetic‐based neuromodulation technology will epitomize its immanent precision and stabilization strengths, gradually being admitted by patients with NDs, especially for those with hereditary or chronic progressive diseases.

### 4.3 Therapeutic Effect Enhancement and Indication Expansion

Arguably, therapeutic effect enhancement and indication expansion will endow the future TSPCI of NDs with prominent competitive advantages in the clinic/market. In detail, the exploration of novel targets should first be highlighted, as the relentless march of pathological mechanism researches are continually renewing our knowledges about NDs. Take PD as an instance, based on the loss of dopaminergic neurons in substantia nigra, DBS targeted at basal ganglia circuits (typically including subthalamic nucleus, globus pallidus internal, and ventralis intermedius nucleus) has been proven to effectively meliorate serious tremor and rigidity muscles for middle and later stage patients.^[^ ^124^ ^]^ While long‐term follow‐up studies demonstrated that it was of no avail for gait disturbance, neither reduced the incidence of complications related to cognitive or emotional disorders, such as dementia and depression.^[^ ^125^ ^]^ Notably, although the dyskinesia of PD roots in the inordinate inhibition of subcortical structures (basal ganglion, thalamus) to the motor cortex, downstream pathways including internal capsule, brain stem, and spinal cord cooperate intimately to accomplish motor reflex,^[^ ^126^ ^]^ and the motor coordination depends on cortex‐cerebellum connection for repeat comparison and adjustment between cortical launch and downstream execution.^[^ ^127^ ^]^ Moreover, there are intricate projections from basal ganglion to brain stem, which may account for the gait disturbance of PD.^[^ ^128^ ^]^ Consequently, exploring novel targets such as cortical somatostatin interneurons,^[^ ^129^ ^]^ midbrain neurons,^[^ ^130^ ^]^ and the cerebellar Purkinje cells,^[^ ^131^ ^]^ could probably further enhance the therapeutic effects of DBS, ameliorating the life quality of advanced PD patients.

Nevertheless, NDs are not limited within several well‐known diseases such as epilepsy and PD, but include a myriad of others related to the damage or dysfunction of neural circuits, for instance, AD, neuropathic pain, neural system infection, demyelination, among which many of them could be intractable for existing treatments. Therefore, it is of great significance to expand the potential applications of TSPCI, to accelerate its clinical translation paces, and meet the ever‐increasing clinical demands.

### 4.4 Industrialization

Industrialization is not only fundamental for clinical transformation, but essential for lowing costs upon technological development and professional staff training, providing affordable products for a tremendous member of NDs patients. Totally different from small‐scaled fabrication in the laboratory, mechanized auxiliary equipment is indispensable for large‐scale industrialized production. For instance, single/two‐screw extruder, internal mixer, and open mill, might be required for polymer material processing,^[^ ^132^ ^]^ during which whether the drugs or other bioactive constituents would be deactivated by the high temperature is a critical point. Moreover, suitable indexes should be adopted for rigorous quality control for raw material selectivity, producing process monitoring, and end‐of‐line product test, to ensure that the biosafety, validity, and specifically the temporal‐spatial precision for personalization, would not be attenuated by large‐scale production. Simultaneously, in most circumstances, the processing parameters in the laboratory might be inapplicable for amplified production, meaning that the scale for industrialized trails should be enlarged step by step for elaborate optimization. Importantly, Good Manufacturing Practice (GMP) from FDA provides essential guidance for standardized production of medical devices, which emphasizes that normative production process guarantees satisfactory quality of products.

In summary, despite the existing challenges, temporal‐spatial precision represents an irresistible trend for the clinical intervention of NDs. As a frontier cross‐disciplinary field, TSPCI is attracting increasing attentions and concerted efforts from neurobiology, bioengineering, chemical materials, and AI, which have developed smart systems with promising clinical values, and proposed plentiful enlightening innovations for further researches. With the emphasis on temporal‐spatial precision, the guarantee of biocompatibility and biosafety, the enhancement of therapeutic effects, the prolong of durable services, as well as the prompt of scaled and high‐quality production, it is foreseeable that in the near future increasingly advanced TSPCI systems would be translated into clinical practice, to support billions of patients plagued with NDs.

## Conflict of Interest

The authors declare no conflict of interest.

## Data Availability

The data that support the findings of this study are available from the corresponding author upon reasonable request.
